# Cannabis Use Patterns Among Adults Living With Chronic Pain Before and During the COVID Pandemic: Insights From the COVID-19 Cannabis Health Study

**DOI:** 10.1155/prm/9631487

**Published:** 2025-10-23

**Authors:** Amrit Baral, Denise C. Vidot, Bria-Necole A. Diggs, Isabella Jimenez, Varan Govind, Eva Widerstrom-Noga, Michelle Weiner, Johis Ortega, Marvin Reid, Jacqueline Sagen

**Affiliations:** ^1^Department of Mental Health, Bloomberg School of Public Health, Johns Hopkins University, Baltimore, Maryland, USA; ^2^School of Nursing and Health Studies, University of Miami, Coral Gables, Florida, USA; ^3^Department of Public Health Sciences, University of Miami Miller School of Medicine, Miami, Florida, USA; ^4^Department of Radiology, University of Miami Miller School of Medicine, Miami, Florida, USA; ^5^The Miami Project to Cure Paralysis, Department of Neurological Surgery, University of Miami Miller School of Medicine, Miami, Florida, USA; ^6^College of Osteopathic Medicine, Nova Southeastern University, Fort Lauderdale, Florida, USA; ^7^Faculty of Medical Sciences, University of the West Indies, Mona, Kingston, Jamaica

**Keywords:** cannabis, chronic pain, COVID, marijuana, pain

## Abstract

**Background:**

This study aims to identify sociodemographic factors associated with cannabis use for chronic pain management before and after COVID-19 was declared a pandemic. Furthermore, it seeks to compare cannabis use patterns in adults with and without chronic pain.

**Methods:**

We analyzed US-based responses from the COVID-19 Cannabis Health Study, a cross-sectional online survey administered via REDCap between March 2020 and March 2022. All respondents were cannabis consumers in the past year. Cannabis use patterns and chronic pain were self-reported via the COVID-19 Cannabis Health Questionnaire. Statistical analysis included Chi-square tests, Fisher's exact tests, *t*-tests, and multivariable logistic regression with a two-tailed alpha of 0.05 for significance.

**Results:**

Among 2243 participants, 50.3% consumed cannabis to manage chronic pain. Younger age (< 40 years; aOR: 3.20, 95% CI: 2.59–3.96), Hispanic/Latino ethnicity (aOR: 2.20, 95% CI: 1.56–3.05), and higher income levels (> $100,000 annually; aOR: 1.69, 95% CI: 1.25–2.29) were associated with higher odds of consuming cannabis to manage chronic pain. Participants using cannabis for chronic pain were more likely to use a CBD/THC ratio. The pandemic led to increased dosages and changes in consumption methods: 40.5% increased their cannabis dose, smoking as the primary method declined from 62.2% before the pandemic to 34.5% afterward, while edibles rose from 7.9% to 30.9%, and tinctures from 3.2% to 8.6%. Route changes varied with chronic pain status.

**Conclusion:**

There was a shift from smoking to nonsmoking methods to manage chronic pain. Those who were younger and those of Hispanic/Latino ethnicity had higher odds of using cannabis for chronic pain.

## 1. Introduction

Chronic pain is a prevalent and debilitating condition affecting a substantial proportion of the adult population. In the United States, it is estimated that between 20.4% and 30.7% of adults suffer from chronic pain, with 8.0% experiencing high-impact chronic pain [1, 2]. Women, older adults, and those with lower socioeconomic status are disproportionately affected by this condition. Chronic pain not only reduces quality of life and limits daily activities but also contributes to increased healthcare costs [1–3]. Chronic pain management traditionally relies on pharmacological treatments, including opioids and nonsteroidal anti-inflammatory drugs (NSAIDs), and adjunct therapies such as physical therapy and massage [4, 5]. However, the long-term use of pharmaceutical treatments is often associated with significant adverse effects, such as gastrointestinal issues, dependency, and potential misuse, leading to a growing interest in alternative therapies [6–8].

In recent years, cannabis has emerged as a promising alternative for pain management, driven by increasing evidence of its analgesic properties [9]. Cannabis contains bioactive compounds called cannabinoids, such as tetrahydrocannabinol (THC) and cannabidiol (CBD), which interact with the body's endogenous cannabinoid system to modulate pain and inflammation [10–12]. THC's analgesic effects are mainly mediated by CB1 receptors, while its immunomodulatory properties involve CB2 receptors [13]. Studies indicate that the use of medicinal cannabis is associated with a reduction in the use of other pain medications, including opioids, which has significant implications for public health, given the opioid crisis [14–17]. Furthermore, patients using cannabis for pain management have reported benefits such as improved sleep and overall quality of life, with relatively few short-term adverse effects, making it a viable option for those with chronic pain [18, 19]. While cannabis shows promise for pain management, research indicates that it can impact cognitive function, with acute intoxication impairing memory, attention, and motor control and prolonged heavy use linked to cognitive deficits, mood disorders, and psychosis [20–22]. The severity of impairment depends on factors such as age of initiation, frequency, and duration of use, though research on long-term effects remains inconclusive due to methodological limitations [23]. Therefore, psychological assessment in managing chronic pain and understanding the reciprocal effects of mental health and cannabis use is crucial. Nevertheless, cannabinoid-based medications show potential for treating various medical conditions [24].

The COVID-19 pandemic had a profound impact on healthcare systems and individual health management practices worldwide, leading to self-management behaviors, particularly in individuals with chronic conditions [25–27]. The pandemic's unprecedented challenges, including increased stress, anxiety, and limited access to traditional healthcare services, have exacerbated the difficulties individuals with chronic pain face [28]. As a result, there has been a notable shift toward alternative treatments, including the use of cannabis for pain relief, anxiety, and depression [29, 30]. This increased trend in cannabis consumption has been partly attributed to the pandemic-induced disruptions in healthcare delivery and the heightened need for self-management strategies [29–31]. While prior research has extensively covered general trends in cannabis use, the specific effects of the pandemic on cannabis use patterns for chronic pain management remain underexplored. This context provides a unique opportunity to investigate how a global public health crisis can alter consumption behaviors, patterns of use, and the adoption of alternative therapies such as cannabis for pain management.

This study aims to address gaps in the literature by examining cannabis use for chronic pain management during the COVID-19 pandemic and identifying key sociodemographic factors associated with its use. Furthermore, it seeks to compare usage patterns between those with and without chronic pain, exploring how the pandemic's unique conditions have influenced these behaviors in both groups. The findings are anticipated to offer valuable insights for healthcare providers, policymakers, and researchers, aiding in the development of targeted public health strategies and therapeutic guidelines for managing chronic pain amid ongoing and future healthcare challenges.

## 2. Methods

### 2.1. Data Source and Study Participants

The data for this analysis were derived from the COVID-19 Cannabis Health Study (CCHS), an investigator-initiated, online cross-sectional survey with recruitment via social media (Twitter, LinkedIn, Facebook, and/or Instagram), community partners, Listservs, email databases, and word of mouth to enable safe distribution amid the pandemic [32–34]. The study aimed to investigate the impact of the COVID-19 pandemic on cannabis use patterns and related behaviors among adults using cannabis for medicinal or adult/recreational purposes. Data collection was managed using REDCap, a secure data management platform hosted at the University of Miami. This software facilitated the prevention of duplicate responses, maintained participant anonymity, monitored timestamps, safely stored survey responses, and ensured data integrity. The study received ethical approval from the University of Miami Institutional Review Board (IRB number: 20200307). Informed consent was obtained from all participants before survey administration.

For this analysis, US-based responses from 2,243 participants collected between March 2020 and March 2022 were extracted from the CCHS dataset. The study included participants aged 18 or older who reported past-year cannabis use and indicated whether they used it for chronic pain management. The overall survey completion rate was 77%. The survey was available only in English and had no restrictions based on participants' country of residence. Participation was voluntary, and no monetary incentives were provided. Before formal data collection, the survey was piloted with five individuals to refine the survey link and gather feedback [32–34].

### 2.2. Measures

All the data collected were self-reported. The sociodemographic variables collected were age, gender, sexual orientation (LGBTQ status), race/ethnicity, income, and educational attainment. Additionally, participants provided information on their cannabis use patterns. This included data on cannabis use within the past 30 days, the purpose of cannabis use (medical-only, recreation/adult-only, both), whether cannabis was recommended by a healthcare provider (yes/no), the dominant cannabinoid present in the cannabis used, and the frequency of being under the influence of the psychoactive effects of THC for six hours or more (yes/no). Participants also reported whether they had increased their cannabis dosage during the pandemic (yes/no) and the routes of cannabis consumption before and after the onset of the pandemic. Furthermore, participants were asked if they use cannabis for the management of chronic pain, with response options being either yes or no. We divided our data collection timeline into two phases of the COVID-19 pandemic: prevaccination availability (March 21, 2020–December 31, 2020) and postvaccination availability (January 1, 2021–January 14, 2022).

### 2.3. Data Analysis

Descriptive statistics were utilized to summarize the sociodemographic characteristics and cannabis use patterns of the analytical sample, both overall and stratified by cannabis use status for managing chronic pain (yes/no). Chi-squared tests or Fisher's exact tests were employed to compare proportions, while *t*-tests were used to compare means, as appropriate. The results were presented as means with standard deviations for numerical variables and as percentages for categorical variables. Multivariable logistic regression analysis was conducted to assess the association between sociodemographic factors and the use of cannabis for chronic pain management. The phases of the pandemic (i.e., pre- and postvaccination) were also adjusted for in the analysis. Adjusted odds ratios (aORs) and 95% confidence intervals (CIs) were reported for the association between each sociodemographic variable and cannabis use for chronic pain management. All statistical analyses were performed using SAS Analytics Version 9.4 (SAS Institute, Inc., Cary, NC, USA), with a significance level set at a two-tailed alpha of 0.05.

## 3. Results

### 3.1. Sociodemographic Characteristics

A total of 2,243 participants were included in the analysis, with 50.3% reporting using cannabis for chronic pain (Table 1). The mean age of the overall sample was 43.3 years (SD = 15.9). Those who reported consuming cannabis to manage chronic pain were older (48.1 years, SD = 14.6) compared to those not consuming cannabis to manage chronic pain (38.5 years, SD = 15.8) (*p* < 0.0001). When categorized by age, 46.3% of participants were under 40 years, and 53.7% were 40 years or more. Among those consuming cannabis for chronic pain, 32.0% were under 40, while 68.0% were 40 or older. In contrast, among those not consuming cannabis for chronic pain, 60.7% were under 40, and 39.3% were 40 or older. These differences were statistically significant (*p* < 0.0001).

Among the participants, 48.9% were female, 49.7% were male, and 1.4% identified as other. The distribution of gender was similar between those with and without cannabis consumption for chronic pain (*p* = 0.60); 19.0% of the sample identified their sexual orientation as LGBTQ, with no significant difference in proportion between those with and without cannabis consumption for chronic pain (*p* = 0.38). In terms of race/ethnicity, 76.1% identified as non-Hispanic White, 4.9% as non-Hispanic Black, 13.6% as Hispanic/Latino, and 5.4% as other. There were significant differences in racial/ethnic distribution between those who did and did not use cannabis for chronic pain (*p* < 0.0001). Non-Hispanic White participants were more prevalent among those with chronic pain (83.0%) that those without chronic pain (69.0%), while non-Hispanic Black participants and Hispanic/Latino participants without chronic pain (6.4% and 19.2%, respectively) were more prevalent than those with chronic pain (3.4% vs. 8.1%, respectively). See Table 1 for details.

Income levels varied significantly between the groups (*p*=0.004), with 27.6% of those using cannabis for chronic pain earning less than $30,000 annually, compared to 23.3% of those not using cannabis for chronic pain. Education levels also differed significantly (*p*=0.003), with 47.2% of chronic pain users reporting some college or technical education, versus 41.1% among nonchronic pain users. While these differences were statistically significant, the absolute differences were modest and may not reflect substantial demographic disparities between the groups.

### 3.2. Cannabis Use Patterns

Of the overall sample, 96.4% reported cannabis use within the past 30 days, with notable differences between those who endorsed cannabis use to manage chronic pain and those who did not (*p* < 0.0001; Table 2). Medical-only use of cannabis was more prevalent among those using cannabis to manage chronic pain (28.9%) compared to those not using it for chronic pain (9.4%). Conversely, recreational-only (adult) use in the past 30 days was more common among those not using cannabis for managing chronic pain (38.6%) than among those who did (6.5%). Overall, 54.7% of participants reported past 30-day use for both medical and recreational purposes, with dual-purpose use more prevalent among chronic pain users (62.7%) than nonusers (46.7%).

A significantly higher proportion of participants who use cannabis to manage chronic pain (62.1%) reported receiving a cannabis recommendation from a healthcare provider than those who did not use cannabis to manage chronic pain (47.2%; *p* < 0.0001). THC-dominant cannabis was the most reported type among all participants (61.9%), with a higher prevalence among those not using it to manage chronic pain (67.4%) compared to those who reported using it for chronic pain (58.4%; *p* < 0.0001). Use of a CBD/THC ratio product was more common in those consuming cannabis for managing chronic pain as compared to those not consuming it to manage chronic pain (31.1% vs. 20.0%, *p* < 0.0001).

### 3.3. Impact of COVID-19 on Cannabis Use Patterns

The pandemic was associated with changes in cannabis use patterns, with 40.4% of participants reporting an increased dose during the pandemic. There was no significant difference between those using cannabis for managing chronic pain and their counterparts regarding the increase in cannabis dosage (*p*=0.17).

**Figure 1:**
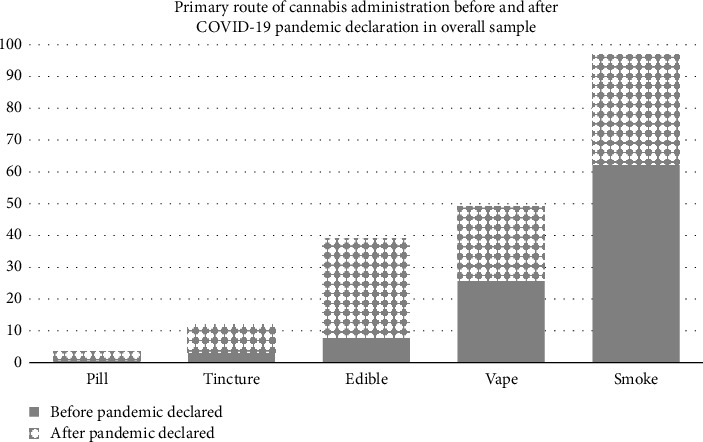
Route of cannabis consumption before and after pandemic was declared in overall sample, COVID-19 Cannabis Health Study, 2020–2022.

Figure 1 illustrates the self-reported routes of cannabis consumption in the overall sample, before and after the pandemic was declared. Before the pandemic, 62.2% of participants reported smoking as their primary method of consumption, but this dropped to 34.5% after the pandemic began. Vaping was reported by 26.0% of participants prepandemic, decreasing slightly to 23.0% postpandemic. The use of edibles increased significantly, rising from 8.0% before the pandemic to 31.0% afterward. Similarly, the use of tinctures increased from 3.2% to 8.5%, and the use of pills rose from fewer than 0.6% to about 3.0%.

**Figure 2:**
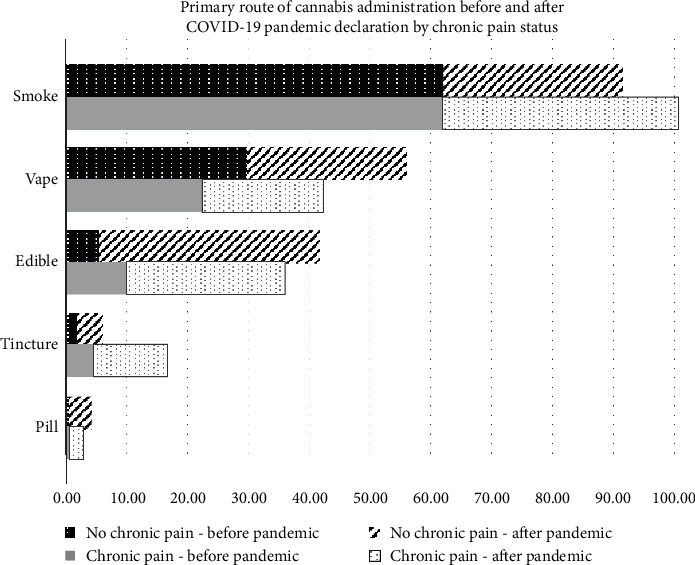
Route of Cannabis consumption in participants, with and without chronic pain subgroups, of the COVID-19 Cannabis Health Study, 2020–2022.

Figure 2 depicts the self-reported routes of cannabis consumption before and after the pandemic was declared, among adults who used cannabis to manage chronic pain and those who did not use cannabis to manage chronic pain. Among those with chronic pain, there was a difference in the prevalence of smoking (62.4% vs. 39.1%), edible (10.0% vs. 26.3%), tincture (4.5% vs. 12.3%), and pill (0.56% vs. 2.2%) use before versus after the pandemic was declared. There were no significant differences in vape use before (22.5%) versus after (20.1%) the pandemic was declared. Among adults who did not use cannabis to manage chronic pain, there was a difference in each route of use before and after the pandemic. Smoking (62.1% vs. 29.4%) and vaping (29.8% vs. 26.3%) decreased after the pandemic. Edible (5.6% vs. 36.3%), tincture (4.4% vs. 1.9%), and pill (3.8% vs. 0.6%) use increased after the pandemic was declared.

### 3.4. Adjusted Odds of Consuming Cannabis for Chronic Pain Management

Multivariable logistic regression analysis revealed several significant associations with cannabis consumption for chronic pain management (Table 3). Participants aged < 40 years had higher odds of consuming cannabis for chronic pain management than those aged > 40 years (aOR = 3.20, 95% CI: 2.59–3.96). Hispanic/Latino participants had higher odds of consuming cannabis for chronic pain management than non-Hispanic White respondents (aOR = 2.20, 95% CI: 1.56–3.05). Participants at the highest income level (> $100,000) had higher odds of cannabis use for chronic pain management than those with the lowest income level (< $30,000) (aOR = 1.69, 95% CI: 1.25–2.29).

## 4. Discussion

Our study explored the prevalence and sociodemographic determinants of cannabis use for chronic pain management during the COVID-19 pandemic. Our results showed that 50.1% of participants reported using cannabis for chronic pain management. While bivariate analyses (Table 1) indicated significant associations between sociodemographic factors: age, race/ethnicity, income, and education level with cannabis use to manage chronic pain, the multivariable regression analysis (Table 3), adjusted for confounders/covariates, including the phases of the COVID-19 pandemic, offered a more nuanced understanding. Although the bivariate analysis suggested higher cannabis use among older age groups and non-Hispanic White participants, multivariable logistic regression results revealed that younger individuals and those of Hispanic/Latino ethnicity had higher odds of using cannabis for chronic pain. Furthermore, while bivariate results indicated a higher prevalence of cannabis use among lower-income groups, the multivariable analysis showed that higher-income individuals had higher odds of consuming cannabis for chronic pain, probably attributed to better access to legal medical cannabis products because of greater financial resources [35, 36]. Age-related trends in cannabis use for pain management vary across studies. Some studies reported that younger adults are more likely to use cannabis for pain, while others documented increasing use among older adults [37–39]. Higher-income individuals are more likely to access medical cannabis programs, which require physician certification and often involve additional costs [40, 41]. However, some studies found a higher concentration of cannabis stores in lower-income neighborhoods [41, 42]. Additionally, neighborhoods with higher racial and ethnic minority populations typically have fewer medical cannabis-certifying providers [43]. This disparity indicates that while physical access to cannabis may be greater in certain areas, financial barriers and limited access to medical authorization remain significant in lower-income and minority groups.

Participants with chronic pain in our study were more likely to use cannabis for medical reasons or both medical and recreational purposes, often choosing CBD and THC-dominant products. Research indicates that individuals with chronic pain are increasingly turning to cannabis as an alternative or adjunct to conventional pain medications [44]. They also reported daily or almost daily use and were more likely to receive recommendations from healthcare providers. In contrast, those not using cannabis for chronic pain were more likely to use it recreationally and preferred THC-dominant products. The study found significant changes in cannabis consumption methods before and after the COVID-19 pandemic. Smoking, which was the predominant method before the pandemic, decreased notably, while the use of edibles increased significantly. Among those using cannabis for chronic pain, there was a notable rise in the use of edibles and tinctures, with a decrease in vaping. Conversely, vaping increased among nonchronic pain users, and the use of cannabis pills rose slightly in both groups after the pandemic began.

Cannabis use patterns and motivations differ among various populations. Research indicates that lower-income individuals and those with chronic pain are more likely to use cannabis for medical purposes, including pain management. This preference may be attributed to the high cost of prescription medications and limited insurance coverage, which make traditional pain treatments less accessible [45–47]. However, in our study, individuals with higher income were more likely to use cannabis for chronic pain management. Additionally, medical cannabis users frequently report preferring cannabis over pharmaceutical drugs, citing better effectiveness, fewer side effects, and lower costs [48]. Although 85.7% of participants using cannabis for pain management in our study were non-Hispanic White, the odds of using cannabis for chronic pain were higher among Hispanic/Latino individuals compared to non-Hispanic White participants. This contrasts with other studies in the United States that often report higher medical cannabis use rates among non-Hispanic White individuals [49]. This discrepancy may stem from differences in study populations, access to medical cannabis, socioeconomic factors, or variations in cultural attitudes toward cannabis.

Our data indicate a significant rise in the use of edibles among those managing chronic pain, suggesting a preference for methods that offer sustained relief without the adverse effects associated with inhalation [44, 50, 51]. This shift toward edibles and tinctures reflects an adaptation to health concerns and a desire for more prolonged pain management [44, 51]. However, it is important to note that edibles come with their challenges, such as delayed onset and unpredictable effects [51]. These findings highlight the need to consider individual preferences and values when discussing medical cannabis options for chronic pain management.

Biologically, the efficacy of cannabis in managing chronic pain is supported by its interaction with the endocannabinoid system (ECS) [12]. THC, the primary psychoactive cannabinoid, binds to CB1 receptors in the central nervous system, potentially altering pain perception and providing analgesic effects [52, 53]. CBD, another key cannabinoid, modulates the ECS and interacts with serotonin and TRPV1 receptors, contributing to its therapeutic effects without THC's psychoactive effects [54, 55]. The observed preference for cannabis products with a balanced THC/CBD ratio and nonsmoking methods may aim to maximize therapeutic benefits while minimizing adverse effects. These findings highlight the need for further research to clarify how different cannabinoids and consumption methods affect pain management and health outcomes.

Our study has several strengths. It addresses a gap in the literature by examining how the COVID-19 pandemic has affected cannabis consumption patterns for chronic pain management. Key strengths include the use of a large, diverse sample from the CCHS, and the complete anonymity provided by the survey, which safeguards participants and ensures genuine responses. The use of REDCap for data collection further ensured data integrity and participant confidentiality. However, the study also has limitations. The cross-sectional design limits causal inferences, and self-reported data may be prone to recall or social desirability bias. The survey's availability only in English may restrict generalizability to non-English–speaking populations. Additionally, while the online format was suitable during the pandemic, it could have led to duplicate responses, although REDCap helped manage this risk. The predominance of non-Hispanic White participants and potential internet access disparities may affect the generalizability of the findings to other racial and ethnic groups. Moreover, the comparison group (non–chronic pain users) includes individuals using cannabis for other medical or recreational reasons, which may mask important differences in use patterns and motivations across these subgroups. Future studies should consider disaggregating these groups to better understand nuances in cannabis use behaviors and clinical relevance. This study used a convenience sample of cannabis users, providing timely insights into use patterns during the pandemic, though generalizability to the broader population is limited. Despite these limitations, the study's methodological rigor, including pilot testing, helps mitigate some potential biases. Additionally, covariates potentially associated with access to cannabis and its use for pain or other reasons, such as US state or country of residence, were not controlled for in our regression analysis.

Our findings have significant implications for public health, clinical practice, and policy. The shift to nonsmoking cannabis methods indicates a growing awareness of the health risks associated with smoking, which should be reflected in public health campaigns and educational efforts. Clinically, the high prevalence of cannabis use for chronic pain underscores the need for healthcare providers to discuss cannabis use with patients, addressing its benefits, risks, and safe consumption practices. Policy-wise, the observed disparities in cannabis use among different racial and ethnic groups highlight the need for equitable access and accurate information regarding risks and benefits. Future research should focus on the long-term effects of the pandemic on cannabis use and the biological mechanisms of cannabis for pain management to guide clinical practices and policy decisions, ultimately improving health outcomes. Furthermore, future studies should include specific information about the type of chronic pain (e.g., time with pain and type of pain) as this may influence the effectiveness of cannabis and, thus, the decision to use it for managing pain.

## 5. Conclusion

In conclusion, younger age, Hispanic/Latino ethnicity, and higher income levels were significant sociodemographic factors driving cannabis use for chronic pain management during the COVID-19 pandemic, potentially highlighting disparities in access to conventional pain treatment options and in reliance on cannabis as an alternative, which should be addressed through healthcare policy. Our findings also point to a growing preference for nonsmoking methods of cannabis use, such as edibles, reflecting both health-conscious choices and interest in longer-lasting pain relief. Given the increased use of cannabis to manage chronic pain, there is a need for further investigation into its long-term effects and mechanisms. The evolving trend of cannabis legalization and normalization, as well as the increasing availability of various cannabis products, calls for rigorous research, evidence-based cannabis literacy, proper patient-provider communication, revised clinical practices, and equitable policies to ensure informed and accessible care.

## Figures and Tables

**Table 1:** Sociodemographic characteristics of participants, with and without chronic pain, who used cannabis in the COVID-19 Cannabis Health Study, 2020–2022.

	Overall cannabis use sample *N* = 2243	Chronic pain subgroup *n* = 1128 (50.3%)	Without chronic pain subgroup *n* = 1115 (49.7%)	*p* value^a^
Age, mean (SD)	43.3 (15.9)	48.1 (14.6)	38.5 (15.8)	**< 0.0001**
Age groups (in years), *n* (%)				**< 0.0001**
18–39 years	1038 (46.3)	361 (32.0)	677 (60.7)	
≥ 40 years	1205 (53.7)	767 (68.0)	438 (39.3)	
Gender, *n* (%)				0.60
Female	1097 (48.9)	540 (47.9)	557 (49.9)	
Male	1114 (49.7)	571 (50.6)	543 (48.7)	
Others	32 (1.4)	17 (1.5)	15 (1.4)	
LGBTQ, *n* (%)	428 (19.1)	207 (18.4)	221 (19.8)	0.38
Race/ethnicity, *n* (%)				**< 0.0001**
Non-Hispanic white	1695 (76.1)	930 (83.0)	765 (69.0)	
Non-Hispanic black	109 (4.9)	38 (3.4)	71 (6.4)	
Hispanic/Latino	304 (13.6)	91 (8.1)	213 (19.2)	
Others	120 (5.4)	61 (5.5)	59 (5.4)	
Income, *n* (%)				**0.004**
Less than $30,000	442 (25.5)	243 (27.6)	199 (23.3)	
$30,000–$50,000	375 (21.6)	200 (22.7)	175 (20.5)	
$50,000–$100,000	518 (29.9)	265 (30.1)	253 (39.6)	
More than $100,000	399 (23.0)	172 (19.5)	227 (26.6)	
Education, *n* (%)				**0.003**
High school or less	235 (10.5)	128 (11.4)	107 (9.6)	
Some college/technical	991 (44.2)	532 (47.3)	459 (41.2)	
Bachelor's degree	591 (26.4)	272 (24.2)	319 (28.6)	
Graduate degree	424 (18.9)	194 (17.2)	230 (20.6)	

*Note:* Bold *p* values denote statistical significance at the 0.05 level.

^a^ *p* values were calculated using Chi-squared test/Fisher's exact test and *t*-tests, where appropriate.

**Table 2:** Cannabis use history and types of cannabinoids used, in participants of COVID-19 Cannabis Health Study, 2020–2022.

	Overall sample *N* = 2243	Chronic pain *n* = 1128 (50.3%)	No chronic pain *n* = 1115 (49.7%)	*p* value
Past 30-day cannabis use category				**< 0.0001**
Recreational use only	503 (22.5)	73 (6.5)	430 (38.6)	
Medical use only	431 (19.2)	326 (28.9)	105 (9.4)	
Medical and recreational use	1226 (54.7)	706 (62.7)	520 (46.7)	
Did not use in past 30-day	80 (3.6)	21 (1.9)	59 (5.3)	
Cannabis recommendation				**< 0.0001**
No recommendation from health provider	415 (41.7)	282 (37.9)	133 (52.8)	
Recommendation from health provider	580 (58.3)	461 (62.1)	119 (47.2)	
Cannabinoid used				**< 0.0001**
CBD dominant	50 (2.9)	40 (3.9)	10 (1.5)	
CBN dominant	3 (0.2)	2 (0.2)	1 (0.2)	
THC dominant	1049 (61.9)	597 (58.4)	452 (67.4)	
CBD and THC ratio	452 (26.7)	318 (31.1)	134 (20.0)	
Other cannabinoid	8 (0.5)	6 (0.6)	2 (0.3)	
Unsure	132 (7.8)	60 (5.9)	72 (10.7)	
≥ Six (6) Hours THC use				**< 0.0001**
Never	268 (12.1)	119 (10.6)	149 (13.5)	
Less than monthly	168 (7.6)	68 (6.1)	100 (9.1)	
Monthly	121 (5.4)	43 (3.9)	78 (7.1)	
Weekly	410 (18.5)	170 (15.2)	240 (21.7)	
Daily/almost daily	1255 (56.5)	718 (64.2)	537 (48.6)	
Cannabis dose increased during pandemic	856 (40.4)	429 (39.0)	427 (41.9)	0.17

*Note:* Bold *p* values denote statistical significance at the 0.05 level.

^a^ *p* values were calculated using Chi-squared test/Fisher's exact test and *t*-tests, where appropriate.

**Table 3:** Adjusted odds of cannabis use for chronic pain by sociodemographic characteristics and COVID-19 pandemic phase.

Variable	Adjusted odds ratio (95% confidence interval)
Age (reference ≥ 40 years)	
Age < 40 years	**3.20 (2.59–3.96)**
Gender (reference = male)	
Female	1.10 (0.88–1.33)
Nonbinary	1.03 (0.44–2.42)
Race/ethnicity (reference = non-Hispanic white)	
Non-Hispanic black	1.52 (0.95–2.42)
Hispanic/Latino	**2.20 (1.56–3.05)**
Others	0.88 (0.56–1.38)
Income (reference = less than $30,000)	
$30,000–$50,000	0.97 (0.72–1.30)
$50,000–$100,000	1.11 (0.84–1.46)
More than $100,000	**1.69 (1.25–2.29)**
Education status (reference = high school or less)	
Some college/technical	0.85 (0.60–1.21)
Bachelor's degree	1.27 (0.87–1.86)
Graduate degree	1.25 (0.83–1.89)
Phases of the pandemic (reference = phase 2 or post vaccination era)	
Phase 1: prevaccination era	0.83 (0.45–1.53)

*Note:* Bold values indicate a statistically significant association.

## Data Availability

The data that support the findings can be made available upon reasonable request to the Principal Investigator, Dr. Denise C. Vidot (dvidot@med.miami.edu).
